# Correlation of neutrophil-lymphocyte ratio and platelet-lymphocyte ratio with serum-ascites albumin gradient in ascites

**DOI:** 10.6026/973206300222445

**Published:** 2026-04-30

**Authors:** Pradeep Nigam, Umesh Pratap Singh, Lokesh Tripathi, Awati Siddhivinayak Rajesh

**Affiliations:** 1 Department of Gastroenterology, Shyam Shah Medical College and Associated Hospitals, Rewa Madhya Pradesh, India; 2 Department of General Medicine, Shyam Shah Medical College and Associated Hospitals, Rewa Madhya Pradesh, India; 3 Department of Pathology, Shyam Shah Medical College and Associated Hospitals, Rewa Madhya Pradesh, India

**Keywords:** Ascites, serum ascites albumin gradient, neutrophil-lymphocyte ratio, platelet-lymphocyte ratio, portal hypertension, inflammatory markers

## Abstract

Differentiating portal hypertensive from non-portal hypertensive ascites remains a common clinical challenge, especially in settings where rapid yet cost-effective diagnostic tools are limited. Serum-ascites albumin gradient (SAAG) is the standard method for etiological classification, but simple adjunctive markers may improve early assessment. Neutrophil-to-lymphocyte ratio (NLR) and platelet-to-lymphocyte ratio (PLR) are inexpensive inflammatory indices with potential diagnostic value. Hence, this cross-sectional study included 100 adult patients with clinically and radiologically confirmed ascites between January and December 2024. Patients were classified into high SAAG (≥1.1 g/dL) and low SAAG (<1.1 g/dL) groups. High SAAG was present in 66% of patients. NLR showed a significant moderate positive correlation with SAAG (r = 0.55; P < 0.001), while PLR showed a weak but significant inverse correlation (r = -0.25; P = 0.0004). NLR >2 demonstrated higher sensitivity (87.9%) and diagnostic accuracy (76.0%), whereas PLR <90 showed higher specificity (64.7%) and positive predictive value (80.3%). ROC analysis showed moderate diagnostic performance for NLR (AUC = 0.72) and PLR (AUC = 0.71). Thus, we show that NLR and PLR may serve as useful non-invasive adjuncts to SAAG for evaluating ascites, particularly in resource-limited settings.

## Background:

Ascites, defined as pathological fluid accumulation in the peritoneal cavity [1], is a common clinical manifestation of hepatic and extrahepatic disorders detectable by ultrasonography or computed tomography [2]. It is broadly classified into cirrhotic (portal hypertension-related) and non-cirrhotic etiologies, with severity ranging from asymptomatic accumulation to abdominal distension and impaired mobility. Chronic hepatic schistosomiasis may lead to periportal fibrosis, portal hypertension, and ascites [3], while tuberculous peritonitis remains an uncommon cause [4]. Malignant ascites affects 3.6-6% of palliative care patients and carries a poor prognosis, requiring comprehensive diagnostic evaluation including laboratory markers and imaging [5, 6, 7, 8- 9]. Inflammatory indices such as neutrophil-lymphocyte ratio (NLR) and platelet-lymphocyte ratio (PLR) have demonstrated clinical utility as cost-effective markers [10, 11]. Regional variations in cirrhosis etiology are reported in rural and Indian populations, with alcohol significantly influencing outcomes [12, 13-14]. Systematic evaluation of ascites reveals diverse underlying causes, and management remains challenging in resource-limited settings [15, 16].

Non-cirrhotic causes also include nephrotic syndrome [17]. Evidence-based management includes sodium restriction and diuretic therapy, while serum-ascites albumin gradient (SAAG) effectively differentiates portal hypertensive ascites [18, 19]. NLR has also been associated with survival prediction and diagnosis of infections including spontaneous bacterial peritonitis [20, 21-22]. Therefore, it is of interest to evaluate the correlation of NLR and PLR with SAAG in patients with ascites and to assess their usefulness as simple, cost-effective adjunctive markers in etiological differentiation.

## Materials and Methods:

## Aim:

To evaluate the correlation between Neutrophil-Lymphocyte Ratio (NLR) and Platelet-Lymphocyte Ratio (PLR) with the Serum Ascites Albumin Gradient (SAAG) in patients presenting with ascites.

## Objectives:

This study aims to assess the association between NLR and SAAG in patients with ascites, assess the association between PLR and SAAG in patients with ascites, compare hematological parameters across different SAAG categories, and explore the potential utility of NLR and PLR as non-invasive indicators for differentiating the etiology of ascites.

## Study design:

A hospital-based, observational cross-sectional study.

## Study setting:

This study was conducted in the Department of General Medicine at Shyam Shah Medical College and its affiliated Sanjay Gandhi Memorial Hospital, Rewa, Madhya Pradesh, India.

## Study duration:

The study was conducted over a 12-month period from January 2024 to December 2024.

## Sample size:

To detect a moderate correlation (r = 0.30) between hematological indices (NLR, PLR) and SAAG with 95% confidence and 80% statistical power, the required sample size was calculated using the formula for correlation studies. The estimated minimum sample size was 85. To account for potential dropouts and incomplete data, a total of 100 patients were enrolled in the final analysis.

## Ethical considerations:

The study was approved by the Institutional Ethics Committee of Shyam Shah Medical College, Rewa (IEC No: IEC/MC/2023/31026). Written informed consent was obtained from all participants before enrollment. All procedures were conducted in accordance with the Declaration of Helsinki and relevant national ethical guidelines.

## Inclusion and Exclusion criteria:

The inclusion criteria for this study encompassed patients aged eighteen years or above with clinically and radiologically confirmed ascites. Patients were excluded if they were below eighteen years of age, pregnant, had known bleeding diathesis, were receiving antibiotic prophylaxis, or were critically ill or non-communicative and thus unable to provide informed consent.

## Data collection:

Demographic and clinical data were recorded using a pre-designed case record form. History, physical examination findings, and relevant comorbidities were noted. Blood and ascitic fluid samples were obtained on the same day.

## Laboratory methods:

Neutrophil-Lymphocyte Ratio (NLR) was calculated by dividing the absolute neutrophil count by the absolute lymphocyte count derived from a complete blood count. Platelet-Lymphocyte Ratio (PLR) was calculated by dividing the platelet count by the absolute lymphocyte count. Serum-Ascites Albumin Gradient (SAAG) was calculated by subtracting the ascitic fluid albumin concentration from the serum albumin concentration, with both measurements performed on the same day.

## Ascetic fluid collection and analysis:

Diagnostic paracentesis was performed under sterile conditions and ascitic fluid was evaluated for albumin, total protein, cytology, and microbiological work-up when indicated.

## SAAG classification:

SAAG was classified into two categories for interpretation. A high SAAG value of 1.1 g/dL or greater was considered indicative of portal hypertension commonly due to cirrhosis or hepatic congestion, while a low SAAG value below 1.1 g/dL was suggestive of non-portal hypertension causes such as malignancy, peritoneal tuberculosis, pancreatitis, or nephrotic syndrome.

## Statistical analysis:

Data were entered in Microsoft Excel and analyzed using SPSS version 25.0 (IBM Corp., Armonk, NY, USA). Quantitative variables were expressed as mean plus or minus standard deviation. Comparisons between two groups were performed using the Independent Student's t-test, while comparisons across more than two groups used one-way ANOVA. The Chi-square test was used for categorical data. Correlation between SAAG and hematological indices was evaluated using Pearson's correlation coefficient. Receiver Operating Characteristic curves were constructed to evaluate the diagnostic performance of NLR and PLR, and parameters such as sensitivity, specificity, positive predictive value, negative predictive value, likelihood ratios, and diagnostic odds ratios were calculated. A p-value less than 0.05 were considered statistically significant.

## Results:

Table 1 (see PDF) presents the baseline demographic characteristics of the 100 patients included in the study. The mean age of participants was 47.77 ± 16.58 years, indicating a wide age range. Among the study population, 60% were male and 40% were female. A majority of patients (61%) belonged to rural areas, while 39% were from urban settings. Socioeconomic status assessment showed that 46% of the patients were from the lower middle class (Class III), followed by 28% from the upper lower class (Class IV), 14% from the lower class (Class V), 10% from the upper middle class (Class II), and only 2% from the upper class (Class I). Regarding lifestyle factors, 38% of patients were identified as alcoholic, whereas the remaining 62% were non-alcoholic, indicating a higher burden of non-alcohol-related ascites in the study population. Among the 62 patients with non-alcoholic ascites, a diverse range of etiologies was identified (Table 2 - see PDF). The most common causes were chronic hepatitis and infectious diseases, each accounting for 22.58% of the cases. Cardiac-related ascites was noted in 19.35% of patients. Malignancy and nephrotic syndrome each contributed to 12.90% of non-alcoholic ascites cases. The remaining 9.67% were classified under miscellaneous causes, reflecting the heterogeneity of ascitic pathophysiology beyond alcohol-related liver disease. Table 3 (see PDF) presents the distribution of the Serum-Ascites Albumin Gradient (SAAG) among the study population. A majority of the patients (66%) demonstrated a high SAAG value (≥1.1 gm/dL), indicative of portal hypertension-related ascites. In contrast, 34% had a low SAAG (<1.1 gm/dL), pointing towards non-portal hypertensive causes such as malignancy, tuberculosis, or nephrotic syndrome. The distribution of Neutrophil-Lymphocyte Ratio (NLR) in relation to Serum-Ascites Albumin Gradient (SAAG) is presented in Table 4 (see PDF). Among patients with low SAAG (<1.1 g/dL), 35.30% had an NLR <1, 17.64% had NLR between 1-2, and 47.06% had NLR >2. In contrast, among those with high SAAG (≥1.1 g/dL), only 3.03% had NLR <1, 9.09% had NLR between 1-2, and a significant majority of 87.88% had NLR >2. Statistical analysis using the Chi-square test revealed a significant association between NLR and SAAG (χ^2^ = 23.107, p < 0.0001). Furthermore, Pearson's correlation coefficient (r = 0.55) indicated a moderate positive correlation between NLR and SAAG, suggesting that higher NLR values were associated with a higher likelihood of portal hypertension-related ascites. The relationship between Platelet-Lymphocyte Ratio (PLR) and Serum-Ascites Albumin Gradient (SAAG) is detailed in Table 5 (see PDF). Among patients with low SAAG (<1.1 g/dL), 35.29% had PLR <90, 47.05% had PLR between 90-210 and 17.65% had PLR >210. In contrast, in the high SAAG (≥1.1 g/dL) group, 74.24% had PLR <90, 22.72% had PLR between 90-210, and only 3.03% had PLR >210. The Chi-square test demonstrated a statistically significant association between PLR and SAAG (χ^2^ = 15.859, p = 0.0004). Additionally, Pearson's correlation coefficient (r = -0.25) indicated a weak negative correlation, suggesting that lower PLR values were more commonly associated with high SAAG, which may reflect a pattern of portal hypertension-related ascites. Table 6 (see PDF) presents the diagnostic performance metrics of Neutrophil-Lymphocyte Ratio (NLR >2) and Platelet-Lymphocyte Ratio (PLR <90) in differentiating high SAAG (≥1.1 g/dL) from low SAAG ascites. NLR (>2) shows high sensitivity (87.9%) and a positive predictive value (PPV) of 78.4%, indicating strong potential as a screening tool for detecting portal hypertension-related ascites. Its negative predictive value (NPV) was 69.2%, with an odds ratio of 8.154 and a correlation coefficient (r) of 0.55, indicating a moderate positive association with SAAG. PLR (<90) had moderate sensitivity (74.2%) but higher specificity (64.7%) and PPV (80.3%), suggesting that it is relatively better for confirming high SAAG. However, its correlation coefficient was -0.25, reflecting a weak inverse relationship with SAAG. Table 7 (see PDF) summarizes the diagnostic performance of NLR and PLR when used individually and in combination for predicting high SAAG ascites. When used individually, NLR (>2) demonstrated higher sensitivity (87.9%) compared to PLR (<90), which had greater specificity (64.7%) and PPV (80.3%). NLR alone showed better overall accuracy (76.0%) and correlation with SAAG (r = 0.55). The "Both Positive" strategy (NLR >2 and PLR <90) yielded the highest specificity (82.4%) and PPV (86.7%), making it ideal for confirming high SAAG. However, sensitivity (59.1%) and NPV (50.9%) were lower, limiting its utility for ruling out disease. The "Either Positive" approach (NLR >2 or PLR <90) achieved the highest sensitivity (97.0%) and NPV (85.7%), along with the lowest negative likelihood ratio (0.085). This makes it highly effective for screening, minimizing missed cases of portal hypertension-related ascites. In conclusion, while "Both Positive" increases diagnostic confidence when present, the "Either Positive" strategy offers superior sensitivity for screening and ruling out high SAAG cases.

Receiver Operating Characteristic (ROC) curves depicting the diagnostic performance of Neutrophil-Lymphocyte Ratio (NLR), Platelet- Lymphocyte Ratio (PLR), both markers being positive, and either marker being positive in predicting high SAAG (≥1.1 g/dL) ascites. NLR demonstrated the highest individual area under the curve (AUC ≈ 0.72), followed by PLR (AUC ≈ 0.71), indicating good diagnostic utility. The combination of both positive markers improved specificity, while the presence of either positive marker maximized sensitivity. The diagnostic performance of Neutrophil-Lymphocyte Ratio (NLR), Platelet-Lymphocyte Ratio (PLR), and their combinations was evaluated using ROC curve analysis. The area under the Curve (AUC) for NLR alone was 0.72, indicating good discriminatory ability in predicting high SAAG ascites (Table 8, Figure 1 - see PDF). PLR showed a comparable AUC of 0.71, suggesting it is also a useful marker. When both NLR and PLR were positive, the AUC remained 0.71, reinforcing their combined utility. However, using an "Either Positive" approach where a positive result was defined by elevation in either NLR or PLR the sensitivity increased significantly, but the AUC dropped to 0.66, indicating reduced specificity. The ROC curves in Figure 1 (see PDF) illustrate the trade-off between sensitivity and specificity for each parameter. Among the individual markers, NLR had the best overall balance. The combined approach of "Both Positive" showed the highest specificity (82.4%) and positive predictive value (86.7%), while the "Either Positive" method offered the highest sensitivity (97.0%) and negative predictive value (85.7%).

## Discussion:

In this cross-sectional study of 100 patients with ascites, neutrophil lymphocyte ratio (NLR) and platelet lymphocyte ratio (PLR) were significantly associated with serum ascites albumin gradient (SAAG). High SAAG (≥1.1 g/dL) was observed in 66% of patients. NLR demonstrated a moderate positive correlation with SAAG (r = 0.55) and high sensitivity (87.9%) for identifying portal hypertension related ascites, whereas PLR showed a weaker inverse correlation (r = -0.25) with comparatively higher specificity (64.7%). The demographic profile, with a mean age of 47.77 ± 16.58 years and male predominance (60%), is consistent with previous studies reflecting the epidemiology of chronic liver disease [10-11]. The predominance of rural patients (61%) further highlights disparities in healthcare access and delayed presentation [12]. Non-alcoholic etiologies accounted for 62% of cases, with chronic hepatitis and infectious causes being most common, indicating a changing disease spectrum in developing regions. High SAAG was present in two-thirds of patients, reaffirming portal hypertension as the principal mechanism of ascites formation [18]. Within this context, inflammatory indices showed clinically meaningful associations. Among patients with high SAAG, 87.9% had NLR >2 compared with 47.1% in the low SAAG group (P < .001), indicating a strong relationship between elevated NLR and portal hypertension related ascites. This finding supports the role of systemic inflammation in cirrhosis, driven by immune dysregulation and bacterial translocation. Similar associations between elevated NLR and disease severity and infection in cirrhosis have been reported previously [19, 20- 21]. PLR demonstrated an inverse association with SAAG, with 74.2% of high SAAG patients having PLR <90 compared with 35.3% in the low SAAG group (P = .0004). This likely reflects thrombocytopenia secondary to hypersplenism in portal hypertension. However, the weaker correlation and lower diagnostic accuracy (71.0%) suggest that PLR has a more limited role as an independent marker. From a diagnostic perspective, NLR showed higher sensitivity (87.9%) and accuracy (76.0%), supporting its use as a screening marker, whereas PLR demonstrated higher specificity (64.7%) and positive predictive value (80.3%), suggesting a role in confirming the diagnosis. The combined use of these markers improved diagnostic performance. The "both positive" approach increased specificity to 82.4% and positive predictive value to 86.7%, whereas the "either positive" approach achieved a sensitivity of 97.0% and negative predictive value of 85.7%, making it particularly useful for ruling out portal hypertension related ascites. Receiver operating characteristic analysis demonstrated moderate discriminative ability for both NLR (AUC, 0.72) and PLR (AUC, 0.71). Similar studies evaluating inflammatory markers in cirrhotic patients have reported comparable ROC-based performance, supporting their clinical applicability [20]. Although these values indicate moderate accuracy, their clinical utility lies in their simplicity, low cost, and widespread availability, particularly in resource-limited settings.

## Limitations:

This study has several limitations. First, its single-center design and relatively small sample size may limit generalizability. Second, the cross-sectional design precludes assessment of causality and temporal changes in inflammatory markers. Third, potential confounding factors, including concurrent infections, medications, and disease severity, were not fully controlled. Finally, external validation of the proposed cutoff values was not performed.

## Conclusion:

NLR and PLR were significantly associated with SAAG in patients with ascites. NLR showed higher sensitivity, whereas PLR showed greater specificity. The combined use of these markers improved diagnostic performance. Thus, we show that NLR and PLR may serve as useful adjuncts to SAAG in differentiating ascites etiology, particularly in resource-limited settings. Further multicenter studies are warranted to validate these findings and establish standardized clinical applications.

## Advancement to knowledge:

This study shows that neutrophil-lymphocyte ratio and platelet-lymphocyte ratio are useful adjunctive markers in the evaluation of ascites. NLR showed higher sensitivity, while PLR showed better specificity for predicting high SAAG ascites. Their combined use improved diagnostic performance. These simple and inexpensive haematological indices may assist early etiological classification, especially in resource-limited settings.
